# Liver transplantation using magnetic anastomosis in pigs

**DOI:** 10.1038/s41598-023-44306-0

**Published:** 2023-11-17

**Authors:** Qiang Lu, Kang Liu, Ai-Hua Shi, Wei Zhang, Yong Wan, Rong-Qian Wu, Yi Lv, Shan-Pei Wang

**Affiliations:** 1 Department of Geriatric Surgery, The First Affiliated Hospital of Xi’an Jiaotong University, Xi’an, Shaanxi Province China; 2 National Local Joint Engineering Research Center for Precision Surgery and Regenerative Medicine, The First Affiliated Hospital of Xi’an Jiaotong University, 277 West Yanta Road, Xi’an, 710061 Shaanxi Province People’s Republic of China; 3 Present Address: Department of Hepatobiliary Surgery, The First Affiliated Hospital of Xi’an Jiaotong University, Xi’an, Shaanxi Province China; 4 National Local Joint Engineering Research Center for Precision Surgery and Regenerative Medicine, The First Affiliated Hospital of Xi’an Jiaotong University, 277 West Yanta Road, Xi’an, 710061 Shaanxi Province People’s Republic of China

**Keywords:** Gastroenterology, Preclinical research

## Abstract

Magnetic anastomosis substantially shortens the duration of vascular anastomosis. We aimed to apply magnetic anastomosis technology (MAT) to donor liver implantations in pig orthotopic liver transplantation (OLT). Twenty healthy adult pigs were randomly divided into donors and recipients, and major vascular anastomosis was performed using MAT during OLT. Recipient liver and kidney function was measured pre-surgery and 12, 24 and 72 h post-surgery. Vascular anastomoses examinations were performed using ultrasound or angiography weekly post-surgery, and pathological examinations of vascular anastomoses were performed during autopsy after animal euthanasia. All recipients survived 24 h after surgery, which is considered as successful transplantation. Anhepatic duration was only 13 min, and no anastomotic obstruction or stenosis, magnetic displacement and anastomotic angulation, or distortion was found upon postoperative examinations of major liver vasculature. Aspartate aminotransferase, alanine aminotransferase, and total bilirubin serum levels increased considerably postoperatively. The follow-up period for this study was 1 year, and the median survival time of all recipients was 115 d (interquartile range = 11–180 d). The main causes of death were liver failure, immune rejection, infection, and arterial anastomotic bleeding. Moreover, vascular anastomoses healed well with a survival time of more than two weeks. We developed a novel magnetic device to create a fast and safe technique to perform major vascular anastomoses in pig liver transplantations. Additionally, the liver graft implantation using MAT considerably shortened the recipient warm ischemia time, which will reduce the extent of ischemia–reperfusion injury. We conclude that MAT is an effective method for donor liver fast implantation in OLT in pigs.

## Introduction

Liver transplantation is an effective method for the treatment of end-stage liver diseases, and the anhepatic phase duration is related to the short- and long-term prognosis of recipients^1,2^. A long anhepatic period is accompanied with long warm ischemia time in donor organs and long blood flow disturbance time in recipient organs, which may increase the risk of allograft dysfunction, intestinal bacterial translocation, acute liver failure, and renal insufficiency.

To the best of our knowledge, the simplest and only readily available option to shorten the anhepatic period is rapid graft revascularization, also called donor liver fast implantation. However, this procedure normally requires a traditional hand-sewn vascular anastomosis. In contrast, magnetic anastomosis technology (MAT) is a convenient and time-saving method for revascularization^3–5^. Previously, MAT has been applied to liver implantation in animal models^4,6,7^. Based on previous research, we developed a novel magnetic device to facilitate a fast and safe technique to perform major vascular anastomoses in pig liver transplantations.

## Results

### Surgical results

The liver transplantation using MAT was successful in all recipient pigs. The median operation time was 225 min (interquartile range [IQR] = 198–375 min), and the median cold ischemia time of donor livers was 59 min (IQR = 40–84 min). Notably, the mean anhepatic duration was only 13 min (IQR = 9–20 min). During the anhepatic phase, the median suprahepatic, infrahepatic inferior vena cava, and portal vein anastomoses times were 4 min (IQR = 3–7), 3 (IQR = 2–5) min, and 3 min (IQR = 2–6) respectively. Additionally, the median recipient warm ischemia time, defined as the time from when the liver graft is taken out of the cold preservative solution to reperfusion, was 4 min (IQR = 3–7 min). However, the hepatic artery and biliary anastomosis using a manual suturing technique seemed time consuming: 17 (IQR = 13–23) min in the hepatic artery anastomosis, and 15 (IQR = 11–20) min in the biliary anastomosis.

### Postoperative results

**Figure 1:**
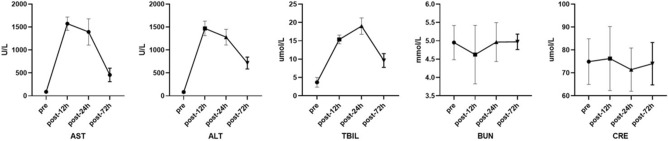
Postoperative liver and kidney function test.

Serum aspartate aminotransferase (AST) and alanine aminotransferase (ALT) levels significantly increased 12 h after operation and then decreased at 24 h postoperatively (AST: pre-surgery 82(IQR = 76–106) U/L, 12 h postoperatively 1538(IQR = 1478–1673) U/L, 24 h postoperatively 1338(IQR = 1193–1596) U/L, and 72 h postoperatively 470(IQR = 309–585) U/L, *p* < 0.05; ALT: pre-surgery 76(IQR = 59–109) U/L, 12 h postoperatively 1463(IQR = 1319–1551) U/L, 24 h postoperatively 1294(IQR = 1128–1390) U/L, and 72 h postoperatively 712(IQR = 622–784) U/L, *p* < 0.05, Fig. 1). However, serum total bilirubin (TBIL) increased continuously from the preoperative level to 24 h postoperatively and then decreased after postoperative 24 h (pre-surgery 3.95(IQR = 2.35–4.45) mmol/L, 12 h postoperatively 15.25(IQR = 14.53–16.10) mmol/L, 24 h postoperatively 19.15(IQR = 17.00–20.98) mmol/L, and 72 h postoperatively 9.40(IQR = 8.38–11.25) mmol/L, respectively, *p* < 0.05, Fig. 1). Notably, there were no significant changes in the BUN and serum CRE levels preoperatively, or 12 h, 24 h and 72 h postoperatively (BUN: pre-surgery 5.05(IQR = 4.51–5.18) mmol/L, 12 h postoperatively 4.40(IQR = 4.01–5.38) mmol/L, 24 h postoperatively 5.06(IQR = 4.71–5.22) mmol/L, and 72 h postoperatively 4.98(IQR = 4.82–5.11) mmol/L, *p* < 0.05; CRE: pre-surgery 73(IQR = 66–86) μmol/L, 12 h postoperatively 80(IQR = 65–86) μmol/L, 24 h postoperatively 70(IQR = 63–80) μmol/L, and 72 h postoperatively 73(IQR = 65–81) μmol/L, *p* < 0.05, Fig. 1).

**Figure 2:**
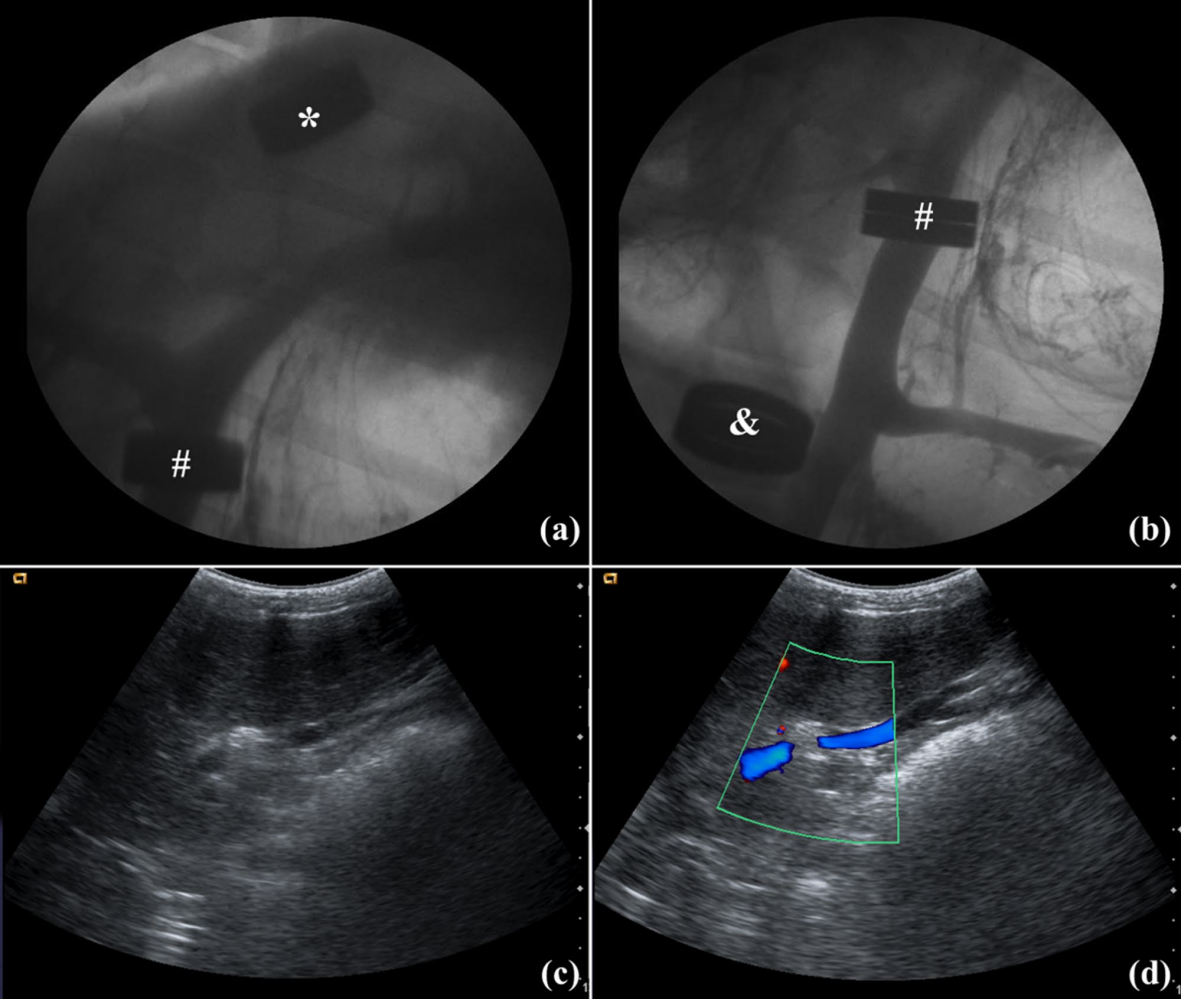
Postoperative anastomotic imaging examination. (**a**), (**b**) Postoperative portal venography: portal vein anastomotic stoma (#), suprahepatic (*) and infrahepatic inferior (#) vena cava anastomotic stoma; (**c**), (**d**) postoperative vascular anastomosis ultrasonography.

The median survival time of recipients after transplantation was 115 (IQR = 11–180) d. The main causes of death in recipients were liver failure (n = 4, 33.3%), immune rejection (n = 3, 25.0%), and infection (n = 2, 16.7%). Other causes of death included pulmonary edema (n = 1, 8.3%) and hepatic artery anastomotic bleeding (n = 1, 8.3%). Additionally, one recipient died of unknown causes. No obstructions or leaks in the inferior vena cava or portal vein inflow were found in recipients immediately after the surgery. No anastomotic obstructions or stenosis, magnetic displacements, or anastomotic angulation or distortion were detected in recipients after transplantation (Fig. 2).

**Figure 3:**
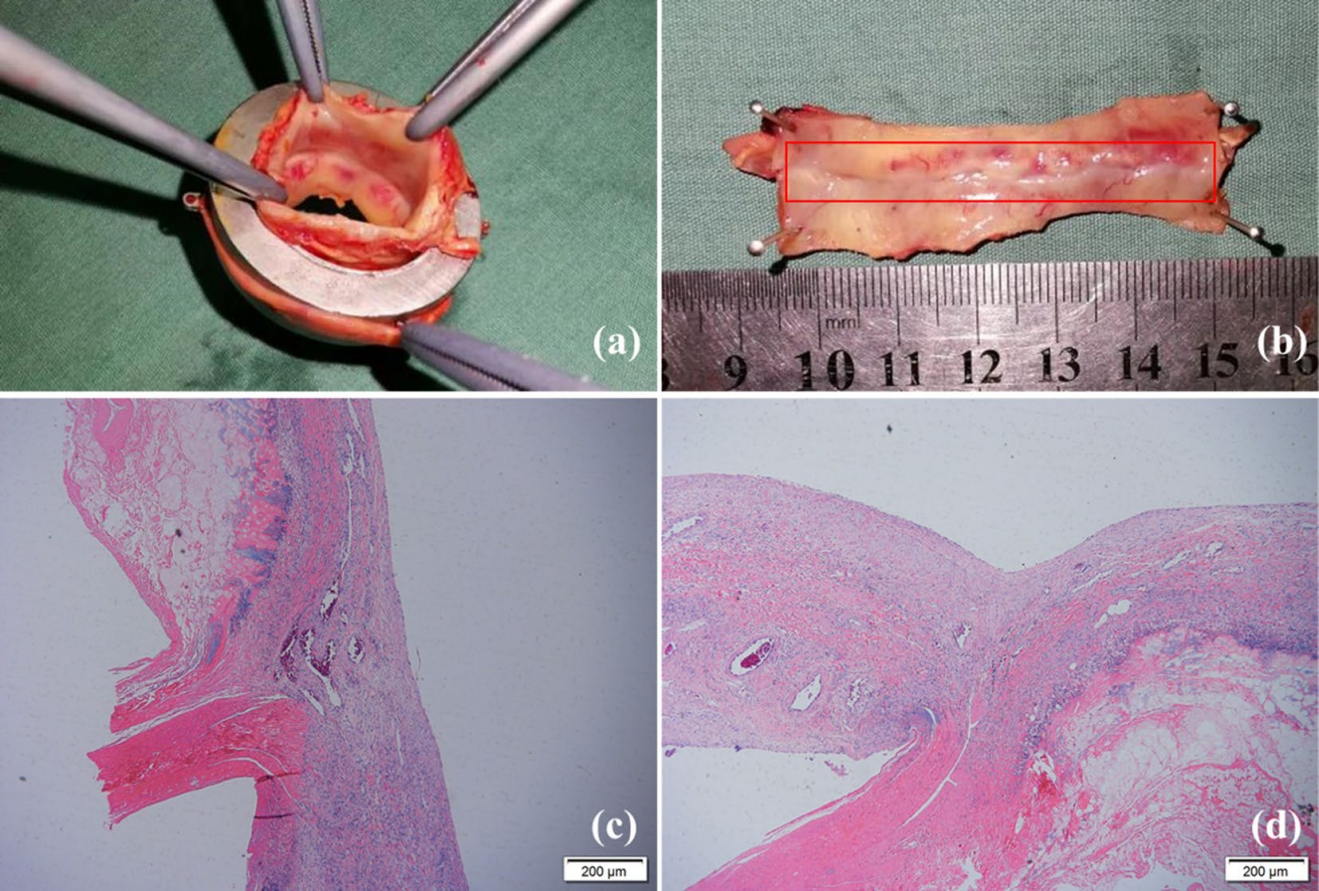
Macroscopic observation and histological examination of the anastomotic stoma (**a**), (**b**) macroscopic observation of the anastomotic stoma; (**c**), (**d**) histological examination of the portal vein anastomotic stoma and infrahepatic inferior vena anastomotic stoma.

Macroscopically, vascular anastomosis using MAT healed in one recipient who then died 13 days after operation. The two magnetic devices had aligned squarely with no evidence of stenosis, stricture or angulation, and the anastomotic stoma surface was smooth with no loss of integrity. Histologically, there was no rough surface or obvious fibrin clot on the surface of the blood vessels. A neat intima-to-intima match was observed, and vascular anastomotic endothelial cells and collagen fibers were arranged regularly (Fig. 3).

## Discussion

In this study, the rapid implantation of donor livers was achieved using MAT in pig liver transplantations, and no anastomotic complications related to MAT were observed postoperatively.

The hand-sewn procedure is still the primary method of vascular anastomosis during liver implantation in clinical liver transplantation, which leads to a longer anhepatic period (40–80 min) because of its complexity and time-consumption^1^. MAT has been successfully applied in the field of digestive tract and fast vascular reconstruction^4–6,8–12^. Unfortunately, the magnetic devices for major vessel reconstruction (vena cava and portal vein) in previous studies are not suitable as permanent implants in organisms. Based on this, we designed this magnetic vascular anastomosis device assembled by a pair of matching Ti-NdFeB composite rings that can be permanently retained in the body. These magnetic anastomosis rings had been successfully applied in end-to-end portal vein anastomosis in previous research and demonstrated good biocompatibility, along with simplifying vascular anastomosis simplified and saving time^12^.

Compared to classical orthotopic liver transplantation, the piggyback procedure resulted in a 50% decrease in the duration of the anhepatic phase, but still requires at least 30 min^13^. In this study, magnetic anastomosis rings were successfully employed in liver implantation, which significantly shortened the anhepatic time (13 min; IQR = 9–20 min). When using MAT, at least a 2-cm vessel stump is required to install the anastomotic ring. Thus, it is essential to dissociate a vascular vessel of sufficient length before the anhepatic phase. In OLT, three major vessels need reconstruction during the anhepatic phase. Unlike that for the portal vein and infrahepatic vena cava, the process of dissociating the suprahepatic vena cava requires some ingenuity due to its special anatomical location. Thus, the phrenic vein was ligated during the process of dissociating the suprahepatic vena cava in this study. However, no diaphragm dysfunction was detected after operation in any recipients.

The inferior vena cava and portal blood flow were blocked during the anhepatic phase of orthotopic liver transplantation, and the intestinal and renal function of the recipient may be impaired due to blood circulation disorders, especially in cases where collateral circulation has not been established, such as in recipients with acute liver failure^14^. Thus, a short anhepatic period may reduce the kidney and intestine damage. In this study, there were no substantial differences in serum BUN or CRE levels before or after the anhepatic phase, implying that the shorter the anhepatic period, the more minor the renal function injury. Additionally, the recipient warm ischemia time, essentially equivalent to the “liver transplant implantation time”, was once considered “fixed time”. Essentially, traditional liver graft implantation technology seems to have no improvement given that modern liver transplant surgical techniques are already generally well sophisticated. However, previous studies indicated that short recipient warm ischemia time might improve short and long-term outcomes after liver transplantation^15–17^. In this study, the liver graft implantation using MAT considerably shortened the recipient warm ischemia time, which will in turn reduce the extent of ischemia–reperfusion injury.

Another issue to consider is the anastomotic angulation or distortion due to mutual magnet attraction and its biocompatibility. The distance between the portal vein anastomosis and inferior hepatic vena cava anastomosis is very short, which may lead to anastomotic angulation or distortion caused by mutual attraction between magnetic devices. However, no anastomotic angulation or distortion was found during postoperative imaging examination in this study. One possible reason for this is that the magnetic force substantially decreases with the increase in distance between the magnets, even when distance increase is small. Another possible reason is that the magnetic ring is wrapped in a magnetic shielding shell, which will prevent mutual attraction between magnets. In this study, each ring contains two parts: the biomedical titanium alloy case and the magnetic core of the NdFeB magnet ring; the titanium is biocompatible and safe. Additionally, no tissue rejection was detected in the histological examination.

There were several limitations in this study. First, this study established the feasibility for magnetic vascular anastomosis in pig liver transplantation; however additional experiments with long-term follow-up are required to further evaluate and verify our findings before human clinical trials can commence. Second, as previous studies have found that magnetic fields may affect cell proliferation, differentiation, and migration, the magnetic field produced by the magnetic devices in this study may have affected the healing of vascular anastomosis and local hemodynamics. Unfortunately, we did not investigate the issue in this study, and further research is warranted.

## Conclusion

We found that MAT is safe and reliable in the rapid implantation of donor livers in pig liver transplantations. AST, ALT, and TBIL serum levels considerably increased post-surgery. The main causes of death were liver failure, immune rejection, infection, and arterial anastomotic bleeding, with median survival time of 115 days. Additionally, the liver graft implantation using MAT considerably shortened the recipient warm ischemia time, which will help reduce the extent of ischemia–reperfusion injury. Therefore, MAT is an effective method for donor liver fast implantation in OLT in pigs.

## Method

### Magnetic device

**Figure 4:**
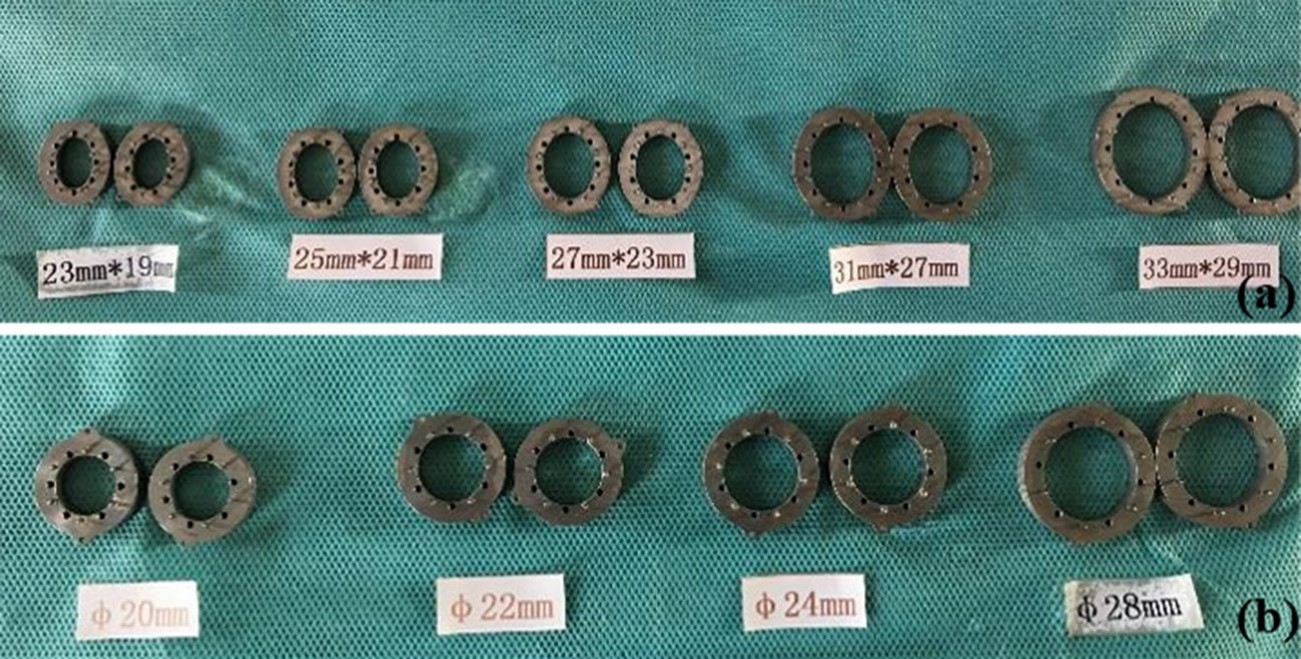
Magnetic device used for vascular anastomosis (**a**) Magnetic anastomosis device for the suprahepatic inferior vena cava; (**b**) Magnetic anastomosis device for the suprahepatic inferior vena cava for infrahepatic inferior vena cava and portal vein.

The magnetic device used in this study has been reported in previous research^5^, and it is composed of a pair of magnetic composite rings. Each magnetic ring has two shapes: circular and ellipse. The circular magnetic rings with inner diameter, outer diameter, and thickness range of 10–18 mm, 20–28 mm, and 5–6 mm, respectively, were used for portal vein anastomosis (Fig. 4a), and the elliptical magnetic rings with the long inner diameter, long outer diameter, short inner diameter, short outer diameter, and thickness range of 13–23 mm, 23–33 mm, 9–19 mm, 19–29 mm, and 5–6 mm, respectively, were used for the anastomosis of the suprahepatic and infrahepatic inferior vena cava. All magnetic devices were sterilized with ethylene oxide before surgery (Fig. 4b).

### Animals

A total of 20 Bama pigs (nine males and 11 females; weight: 60–70 kg), provided by the Experimental Animal Center of Xi'an Jiaotong University, were housed in a pig stable and fed a corn-soybean meal–based diet, and were randomly selected as donors or recipients. This study was approved by the Experimental Animal Management Committee of the Medical Department of Xi'an Jiaotong University (2021-595). All experiments complied with the ARRIVE guidelines and were performed in accordance with the National Institutes of Health guide for the care and use of laboratory animals (NIH Publications No. 8023, revised 1978).

### Study design

Donor and recipient surgeries were performed simultaneously in a shared operating room. The pigs were weighed and then anesthetized using an intravenous injection of 3% pentobarbital sodium solution (1 mL/kg). Implantations were completed within 1 h after donor livers were isolated. Total operation, cold ischemia, vascular anastomosis, biliary anastomosis, anhepatic, postoperative survival times, along with cause of death were recorded.

### Surgical procedure

**Figure 5:**
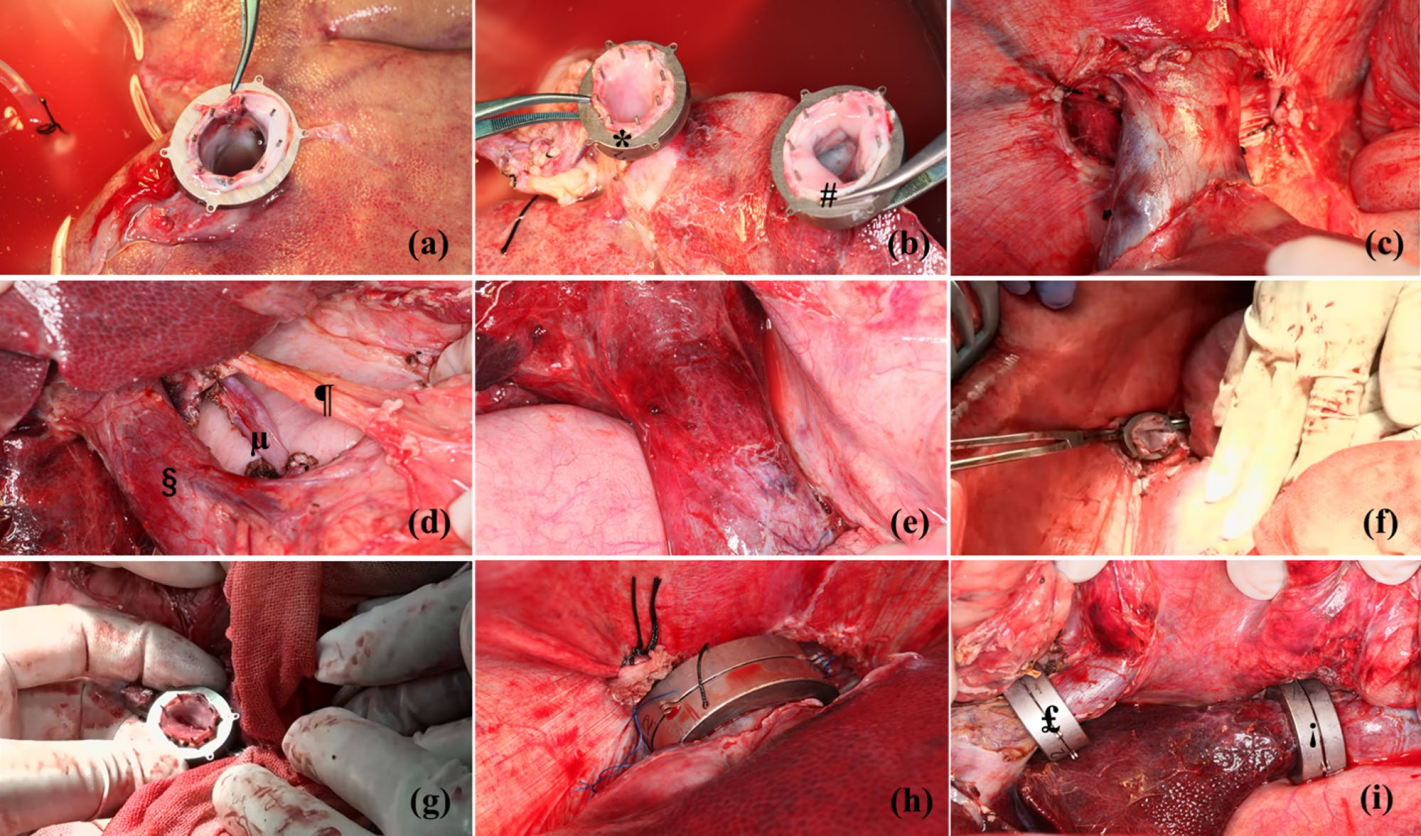
Process of orthotopic liver transplantation (OLT) using magnetic anastomosis technique (MAT). (**a**) Installation of magnetic ring in the suprahepatic inferior vena cava of donor liver; (**b**) installation of a magnetic ring in the infrahepatic inferior vena (#) cava and portal (*) vein of donor liver; (**c**) suprahepatic inferior vena cava of the recipient after dissociating; (**d**) hepatic hilum of the recipient after dissociating: portal vein (§), liver artery (µ), common bile duct (¶); (**e**) infrahepatic inferior vena of the recipient after dissociating; (**f**) installation of a magnetic ring in the suprahepatic inferior vena cava of the recipient; (**g**) installation of a magnetic ring in the infrahepatic inferior vena cava of the recipient; (**h**) suprahepatic inferior vena cava anastomosis using MAT; (**i**) infrahepatic inferior vena cava (¡) and portal vein (£) anastomosis using MAT.

The pigs were euthanized by excessive hemorrhage under full anesthesia after adequate mobilization of the liver. The donor livers were cold flushed with University of Wisconsin (UW) solution, harvested, and then preserved by simple cold storage in UW solution at 4 °C. Next, the portal vein stump was slipped through the internal hole of the magnetic ring and everted onto the pins with equal tension around the circumference. The same operation was performed to the stump of the suprahepatic and infrahepatic inferior vena cava (Fig. 5a, b).

Recipient abdomens were incised through a J-Shaped Incision, and the blood vessels of the liver were fully free by 3 cm. Unlike the traditional liver implantation technique, first in this procedure, the phrenic vein had to be ligated and disconnected to the fully free suprahepatic inferior vena cava by 3 cm (Fig. 5c–e). Then, the vessels were clamped and cut, and the recipient liver was removed. Subsequently, the portal vein and suprahepatic and infrahepatic inferior vena cava were slipped through the internal hole of the magnetic ring and everted onto the pins with equal tension around the circumference in turn (Fig. 5f, g). Then, the donor livers were placed in situ, and the magnetic rings of the donor and recipient vessels were attached to each other immediately for a rapid reconstruction (Fig. 5h, i). Next, the blockage of the portal vein and the suprahepatic and infrahepatic inferior vena cava was sequentially removed for an instant restoration of blood flow. The liver was washed with 43 °C saline to help with rewarming. Finally, an end-to-end hepatic artery anastomosis was performed using a 7–0 Prolene suture by manual suture, and the end-to-end biliary anastomosis was performed using a 5–0 absorbable suture by manual suture.

### Postoperative management

An intravenous drip of 3 mg/kg Cefoperazone Sulbactam Sodium (Pfizer Inc., New York City, USA) was administered daily for three days after the liver transplantation. Intravenous fluids were administered for one day, after which oral hydration was permitted. The animals also received intravenous injections of 1.0 mg/kg flurbiprofen axetil (Beijing Taide Pharmaceutical Co., Ltd, Beijing, China) to relieve pain, but no immunosuppressants were administered.

### Imaging study

An abdominal vascular ultrasound was performed to evaluate the patency of vascular anastomotic stoma immediately, one week, and four weeks after surgery. Transfemoral venography was also employed as an alternative if vascular ultrasound failed (Fig. 2).

### Liver and kidney function tests

Liver and kidney function tests, including AST, ALT, TBIL, BUN, and CRE levels, were performed 1 h before surgery, and 24 h and 72 h after surgery.

### Tissue harvest and histological study

The venous tissues, including the anastomotic site, were sampled for histopathological analysis after the animals were euthanized. All samples were fixed in 10% neutral buffered formalin. Longitudinal paraffin sections were cut at a 5-μm thickness and stained with hematoxylin and eosin. Masson’s trichrome staining was used to differentiate the connective tissue components.

### Statistical analysis

Data are presented as n (%) or median (IQR). Differences between means were assessed using the paired *t*-test or Wilcoxon signed rank test where applicable. *p* < 0.05 was considered statistically significant.

## Data Availability

The original data can be obtained from the corresponding author by email.
